# 4-[(3-Nitro­benzyl­idene)amino]-3-(pyridin-4-yl)-1*H*-1,2,4-triazole-5(4*H*)-thione

**DOI:** 10.1107/S1600536811054444

**Published:** 2012-01-07

**Authors:** Tian-Bao Li, Ming-Sheng Yang, Bang-Shao Yin

**Affiliations:** a College of Chemistry and Chemical Engineering, Hunan Normal University, Changsha, Hunan 410081, People’s Republic of China

## Abstract

In the title compound, C_14_H_10_N_6_O_2_S, the dihedral angle between the pyridine and triazole rings is 3.21 (10)°. The mol­ecule is significantly twisted about the N_t_—N_b_ (t = triazole and b = benzyl­idene) bond [C—N_t_—N_b_=C = 151.64 (17)°]. In the crystal, mol­ecules are linked by weak N—H⋯N hydrogen bonds, generating *C*(8) chains propagating in [10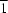].

## Related literature

For further details of the synthesis, see: Wang *et al.* (2010 ▶). For the biological activity of related compounds, see: Liu *et al.* (2011 ▶). 
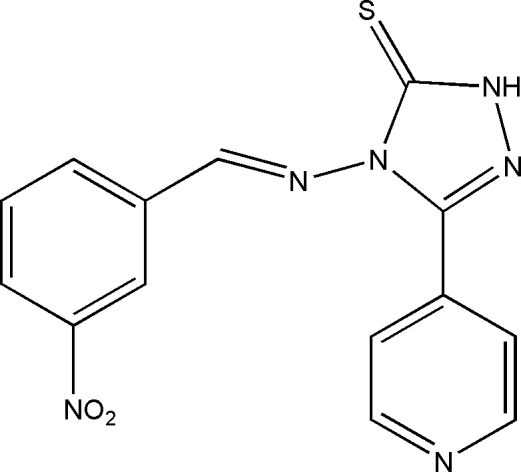


## Experimental

### 

#### Crystal data

C_14_H_10_N_6_O_2_S
*M* *_r_* = 326.34
Monoclinic, 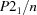

*a* = 3.7989 (13) Å
*b* = 24.334 (9) Å
*c* = 15.208 (6) Å
β = 93.035 (5)°
*V* = 1403.9 (9) Å^3^
*Z* = 4
Mo *K*α radiation
μ = 0.25 mm^−1^
*T* = 113 K
0.20 × 0.18 × 0.10 mm

#### Data collection

Rigaku Saturn724 CCD diffractometer
Absorption correction: multi-scan (*CrystalClear*; Rigaku/MSC, 2005 ▶) *T*_min_ = 0.952, *T*_max_ = 0.975
14478 measured reflections
3317 independent reflections
2807 reflections with *I* > 2σ(*I*)
*R*_int_ = 0.056

#### Refinement

*R*[*F* ^2^ > 2σ(*F* ^2^)] = 0.049
*wR*(*F* ^2^) = 0.103
*S* = 1.07
3317 reflections
212 parameters
7 restraints
H atoms treated by a mixture of independent and constrained refinement
Δρ_max_ = 0.30 e Å^−3^
Δρ_min_ = −0.28 e Å^−3^

### 

Data collection: *CrystalClear* (Rigaku/MSC, 2005 ▶); cell refinement: *CrystalClear*; data reduction: *CrystalClear*; program(s) used to solve structure: *SHELXS97* (Sheldrick, 2008 ▶); program(s) used to refine structure: *SHELXL97* (Sheldrick, 2008 ▶); molecular graphics: *SHELXTL* (Sheldrick, 2008 ▶); software used to prepare material for publication: *SHELXL97*.

## Supplementary Material

Crystal structure: contains datablock(s) gllobal, I. DOI: 10.1107/S1600536811054444/hb6570sup1.cif

Structure factors: contains datablock(s) I. DOI: 10.1107/S1600536811054444/hb6570Isup2.hkl

Supplementary material file. DOI: 10.1107/S1600536811054444/hb6570Isup3.cml

Additional supplementary materials:  crystallographic information; 3D view; checkCIF report

## Figures and Tables

**Table 1:** Hydrogen-bond geometry (Å, °)

*D*—H⋯*A*	*D*—H	H⋯*A*	*D*⋯*A*	*D*—H⋯*A*
N3—H3*A*⋯N1^i^	0.90 (1)	1.96 (1)	2.815 (2)	158 (2)

Symmetry code: (i) 
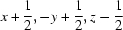
.

**Fig. 1.**
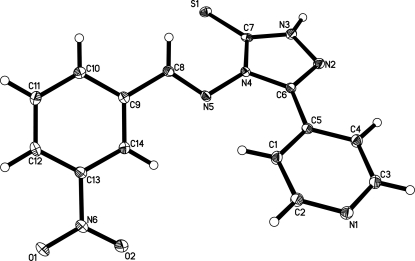
The molecular structure of (I). Displacement ellipsoids are drawn at the 30% probability level.

**Fig. 2.**
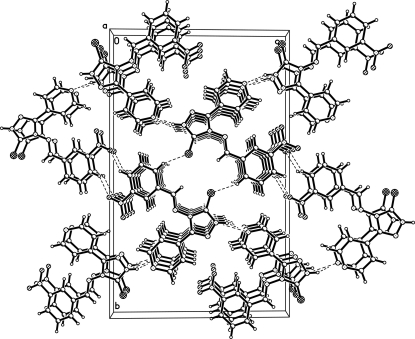
The crystal packing for (I).

## supplementary crystallographic information

### Experimental

The title complex was prepared according to the literature procedures (Wang *et al.* (2010)). Colourless prisms were recrystallised from dimethylformamide solution at room temperature.

### Refinement

All the H atoms were positioned geometrically (C—H = 0.93–0.97 Å) and refined as riding with *U*_iso_(H) = 1.2*U*_eq_(C) or 1.5*U*_eq_(methyl C).

### Crystal data

**Table:**

C_14_H_10_N_6_O_2_S	*F*(000) = 672
*M**_r_* = 326.34	*D*_x_ = 1.544 Mg m^−^^3^
Monoclinic, *P*2_1_/*n*	Mo *K*α radiation, λ = 0.71073 Å
*a* = 3.7989 (13) Å	Cell parameters from 4572 reflections
*b* = 24.334 (9) Å	θ = 1.6–28.0°
*c* = 15.208 (6) Å	µ = 0.25 mm^−^^1^
β = 93.035 (5)°	*T* = 113 K
*V* = 1403.9 (9) Å^3^	Prism, colorless
*Z* = 4	0.20 × 0.18 × 0.10 mm

### Data collection

**Table:**

Rigaku Saturn724 CCD diffractometer	3317 independent reflections
Radiation source: rotating anode	2807 reflections with *I* > 2σ(*I*)
multilayer	*R*_int_ = 0.056
Detector resolution: 14.22 pixels mm^-1^	θ_max_ = 27.9°, θ_min_ = 1.6°
ω and φ scans	*h* = −4→4
Absorption correction: multi-scan (*CrystalClear*; Rigaku/MSC, 2005)	*k* = −32→32
*T*_min_ = 0.952, *T*_max_ = 0.975	*l* = −19→20
14478 measured reflections	

### Refinement

**Table:**

Refinement on *F*^2^	Primary atom site location: structure-invariant direct methods
Least-squares matrix: full	Secondary atom site location: difference Fourier map
*R*[*F*^2^ > 2σ(*F*^2^)] = 0.049	Hydrogen site location: inferred from neighbouring sites
*wR*(*F*^2^) = 0.103	H atoms treated by a mixture of independent and constrained refinement
*S* = 1.07	*w* = 1/[σ^2^(*F*_o_^2^) + (0.0391*P*)^2^ + 0.3217*P*] where *P* = (*F*_o_^2^ + 2*F*_c_^2^)/3
3317 reflections	(Δ/σ)_max_ = 0.002
212 parameters	Δρ_max_ = 0.30 e Å^−^^3^
7 restraints	Δρ_min_ = −0.28 e Å^−^^3^

### Special details

**Table:**

Geometry. All e.s.d.'s (except the e.s.d. in the dihedral angle between two l.s. planes) are estimated using the full covariance matrix. The cell e.s.d.'s are taken into account individually in the estimation of e.s.d.'s in distances, angles and torsion angles; correlations between e.s.d.'s in cell parameters are only used when they are defined by crystal symmetry. An approximate (isotropic) treatment of cell e.s.d.'s is used for estimating e.s.d.'s involving l.s. planes.
Refinement. Refinement of *F*^2^ against ALL reflections. The weighted *R*-factor *wR* and goodness of fit *S* are based on *F*^2^, conventional *R*-factors *R* are based on *F*, with *F* set to zero for negative *F*^2^. The threshold expression of *F*^2^ > σ(*F*^2^) is used only for calculating *R*-factors(gt) *etc*. and is not relevant to the choice of reflections for refinement. *R*-factors based on *F*^2^ are statistically about twice as large as those based on *F*, and *R*- factors based on ALL data will be even larger.

### Fractional atomic coordinates and isotropic or equivalent isotropic displacement parameters (Å^2^)

**Table:**

	*x*	*y*	*z*	*U*_iso_*/*U*_eq_	
S1	0.79238 (13)	0.064958 (19)	−0.05911 (3)	0.01910 (14)	
O1	1.3928 (4)	0.08135 (6)	0.49993 (9)	0.0325 (4)	
O2	1.2099 (6)	0.14915 (7)	0.41988 (12)	0.0631 (7)	
N1	0.0656 (4)	0.30533 (6)	0.24207 (10)	0.0201 (4)	
N2	0.3124 (4)	0.20542 (6)	−0.04085 (10)	0.0176 (3)	
N3	0.4482 (4)	0.16171 (6)	−0.08450 (10)	0.0169 (3)	
N4	0.5138 (4)	0.14148 (6)	0.05152 (9)	0.0144 (3)	
N5	0.6482 (4)	0.12137 (6)	0.13193 (10)	0.0163 (3)	
N6	1.2412 (5)	0.10008 (7)	0.43334 (11)	0.0270 (4)	
C1	0.2990 (5)	0.21757 (8)	0.20265 (12)	0.0203 (4)	
H1	0.3918	0.1830	0.2216	0.024*	
C2	0.2005 (5)	0.25621 (8)	0.26337 (12)	0.0221 (4)	
H2	0.2308	0.2472	0.3241	0.027*	
C3	0.0267 (5)	0.31683 (8)	0.15567 (12)	0.0217 (4)	
H3	−0.0704	0.3515	0.1387	0.026*	
C4	0.1197 (5)	0.28122 (8)	0.09009 (12)	0.0198 (4)	
H4	0.0884	0.2915	0.0299	0.024*	
C5	0.2600 (5)	0.23011 (7)	0.11340 (12)	0.0153 (4)	
C6	0.3606 (5)	0.19291 (7)	0.04253 (11)	0.0152 (4)	
C7	0.5807 (5)	0.12176 (7)	−0.03166 (12)	0.0152 (4)	
C8	0.6464 (5)	0.06903 (7)	0.14201 (12)	0.0160 (4)	
H8	0.5481	0.0453	0.0976	0.019*	
C9	0.8035 (5)	0.04757 (8)	0.22553 (11)	0.0158 (4)	
C10	0.8222 (5)	−0.00903 (8)	0.24024 (12)	0.0176 (4)	
H10	0.7268	−0.0337	0.1968	0.021*	
C11	0.9793 (5)	−0.02948 (8)	0.31800 (12)	0.0205 (4)	
H11	0.9920	−0.0681	0.3271	0.025*	
C12	1.1176 (5)	0.00569 (8)	0.38243 (12)	0.0197 (4)	
H12	1.2262	−0.0081	0.4356	0.024*	
C13	1.0928 (5)	0.06162 (8)	0.36685 (12)	0.0189 (4)	
C14	0.9407 (5)	0.08343 (8)	0.29021 (12)	0.0186 (4)	
H14	0.9297	0.1221	0.2816	0.022*	
H3A	0.461 (5)	0.1634 (9)	−0.1431 (7)	0.025 (6)*	

### Atomic displacement parameters (Å^2^)

**Table:**

	*U*^11^	*U*^22^	*U*^33^	*U*^12^	*U*^13^	*U*^23^
S1	0.0194 (3)	0.0182 (2)	0.0198 (3)	0.00006 (19)	0.00267 (19)	−0.00447 (18)
O1	0.0432 (10)	0.0331 (8)	0.0195 (8)	0.0039 (7)	−0.0132 (7)	0.0013 (6)
O2	0.1174 (18)	0.0204 (8)	0.0460 (11)	−0.0044 (10)	−0.0482 (11)	−0.0003 (7)
N1	0.0243 (10)	0.0202 (8)	0.0160 (8)	0.0027 (7)	0.0030 (7)	−0.0009 (6)
N2	0.0213 (9)	0.0165 (8)	0.0150 (8)	0.0000 (7)	0.0015 (6)	−0.0010 (6)
N3	0.0231 (9)	0.0172 (8)	0.0108 (7)	−0.0001 (7)	0.0029 (7)	−0.0004 (6)
N4	0.0163 (8)	0.0149 (7)	0.0118 (7)	0.0001 (6)	−0.0009 (6)	0.0009 (6)
N5	0.0183 (8)	0.0190 (8)	0.0114 (7)	0.0024 (6)	−0.0017 (6)	0.0005 (6)
N6	0.0354 (11)	0.0249 (9)	0.0196 (9)	0.0001 (8)	−0.0082 (8)	0.0013 (7)
C1	0.0266 (11)	0.0173 (9)	0.0171 (9)	0.0038 (8)	0.0016 (8)	0.0023 (7)
C2	0.0312 (12)	0.0219 (10)	0.0135 (9)	0.0044 (9)	0.0030 (8)	0.0008 (7)
C3	0.0241 (11)	0.0206 (10)	0.0204 (10)	0.0050 (8)	0.0003 (8)	0.0006 (7)
C4	0.0239 (11)	0.0194 (9)	0.0162 (9)	0.0024 (8)	0.0014 (8)	0.0022 (7)
C5	0.0135 (9)	0.0164 (8)	0.0158 (9)	−0.0015 (7)	0.0002 (7)	−0.0003 (7)
C6	0.0155 (10)	0.0153 (8)	0.0144 (9)	−0.0010 (7)	−0.0011 (7)	0.0008 (7)
C7	0.0132 (9)	0.0184 (9)	0.0140 (9)	−0.0054 (7)	0.0012 (7)	−0.0020 (7)
C8	0.0144 (10)	0.0180 (9)	0.0155 (9)	−0.0003 (7)	0.0006 (7)	−0.0003 (7)
C9	0.0143 (10)	0.0187 (9)	0.0146 (9)	0.0008 (7)	0.0020 (7)	0.0010 (7)
C10	0.0168 (10)	0.0182 (9)	0.0180 (9)	0.0014 (8)	0.0037 (7)	−0.0021 (7)
C11	0.0224 (11)	0.0171 (9)	0.0221 (10)	0.0029 (8)	0.0027 (8)	0.0023 (7)
C12	0.0177 (10)	0.0247 (10)	0.0166 (9)	0.0027 (8)	0.0001 (8)	0.0056 (7)
C13	0.0188 (10)	0.0216 (9)	0.0161 (9)	0.0008 (8)	−0.0010 (8)	−0.0007 (7)
C14	0.0213 (11)	0.0170 (9)	0.0174 (9)	0.0006 (8)	−0.0001 (8)	0.0012 (7)

### Geometric parameters (Å, °)

**Table:**

S1—C7	1.6634 (19)	C3—C4	1.381 (3)
O1—N6	1.226 (2)	C3—H3	0.9500
O2—N6	1.216 (2)	C4—C5	1.392 (2)
N1—C2	1.334 (2)	C4—H4	0.9500
N1—C3	1.344 (2)	C5—C6	1.473 (2)
N2—C6	1.308 (2)	C8—C9	1.470 (2)
N2—N3	1.369 (2)	C8—H8	0.9500
N3—C7	1.342 (2)	C9—C14	1.395 (2)
N3—H3A	0.896 (9)	C9—C10	1.396 (3)
N4—C6	1.384 (2)	C10—C11	1.388 (3)
N4—N5	1.389 (2)	C10—H10	0.9500
N4—C7	1.389 (2)	C11—C12	1.383 (3)
N5—C8	1.283 (2)	C11—H11	0.9500
N6—C13	1.468 (2)	C12—C13	1.384 (3)
C1—C2	1.383 (3)	C12—H12	0.9500
C1—C5	1.391 (3)	C13—C14	1.379 (3)
C1—H1	0.9500	C14—H14	0.9500
C2—H2	0.9500		
			
C2—N1—C3	116.43 (16)	N2—C6—N4	110.06 (16)
C6—N2—N3	104.61 (15)	N2—C6—C5	122.55 (16)
C7—N3—N2	114.30 (15)	N4—C6—C5	127.40 (16)
C7—N3—H3A	126.1 (14)	N3—C7—N4	102.37 (15)
N2—N3—H3A	119.3 (13)	N3—C7—S1	128.51 (14)
C6—N4—N5	122.59 (14)	N4—C7—S1	129.03 (14)
C6—N4—C7	108.58 (15)	N5—C8—C9	116.82 (16)
N5—N4—C7	127.12 (15)	N5—C8—H8	121.6
C8—N5—N4	116.80 (15)	C9—C8—H8	121.6
O2—N6—O1	122.78 (18)	C14—C9—C10	119.34 (17)
O2—N6—C13	118.63 (16)	C14—C9—C8	120.42 (17)
O1—N6—C13	118.58 (17)	C10—C9—C8	120.23 (16)
C2—C1—C5	118.91 (18)	C11—C10—C9	120.39 (17)
C2—C1—H1	120.5	C11—C10—H10	119.8
C5—C1—H1	120.5	C9—C10—H10	119.8
N1—C2—C1	124.13 (18)	C12—C11—C10	120.77 (17)
N1—C2—H2	117.9	C12—C11—H11	119.6
C1—C2—H2	117.9	C10—C11—H11	119.6
N1—C3—C4	123.79 (18)	C11—C12—C13	117.85 (17)
N1—C3—H3	118.1	C11—C12—H12	121.1
C4—C3—H3	118.1	C13—C12—H12	121.1
C3—C4—C5	119.07 (17)	C14—C13—C12	123.01 (18)
C3—C4—H4	120.5	C14—C13—N6	117.72 (17)
C5—C4—H4	120.5	C12—C13—N6	119.25 (16)
C1—C5—C4	117.65 (17)	C13—C14—C9	118.64 (17)
C1—C5—C6	124.09 (17)	C13—C14—H14	120.7
C4—C5—C6	118.25 (16)	C9—C14—H14	120.7
			
C6—N2—N3—C7	0.1 (2)	N2—N3—C7—S1	−175.14 (13)
C6—N4—N5—C8	151.64 (17)	C6—N4—C7—N3	−2.68 (19)
C7—N4—N5—C8	−44.9 (2)	N5—N4—C7—N3	−167.98 (16)
C3—N1—C2—C1	−0.1 (3)	C6—N4—C7—S1	174.07 (14)
C5—C1—C2—N1	0.6 (3)	N5—N4—C7—S1	8.8 (3)
C2—N1—C3—C4	−0.4 (3)	N4—N5—C8—C9	177.27 (15)
N1—C3—C4—C5	0.5 (3)	N5—C8—C9—C14	1.1 (3)
C2—C1—C5—C4	−0.5 (3)	N5—C8—C9—C10	−177.83 (17)
C2—C1—C5—C6	178.91 (18)	C14—C9—C10—C11	−0.7 (3)
C3—C4—C5—C1	0.0 (3)	C8—C9—C10—C11	178.18 (17)
C3—C4—C5—C6	−179.44 (17)	C9—C10—C11—C12	0.5 (3)
N3—N2—C6—N4	−1.9 (2)	C10—C11—C12—C13	0.2 (3)
N3—N2—C6—C5	178.17 (16)	C11—C12—C13—C14	−0.7 (3)
N5—N4—C6—N2	169.09 (15)	C11—C12—C13—N6	−179.16 (17)
C7—N4—C6—N2	3.0 (2)	O2—N6—C13—C14	3.3 (3)
N5—N4—C6—C5	−10.9 (3)	O1—N6—C13—C14	−176.44 (19)
C7—N4—C6—C5	−177.05 (17)	O2—N6—C13—C12	−178.2 (2)
C1—C5—C6—N2	178.28 (18)	O1—N6—C13—C12	2.1 (3)
C4—C5—C6—N2	−2.3 (3)	C12—C13—C14—C9	0.4 (3)
C1—C5—C6—N4	−1.7 (3)	N6—C13—C14—C9	178.92 (17)
C4—C5—C6—N4	177.75 (18)	C10—C9—C14—C13	0.3 (3)
N2—N3—C7—N4	1.6 (2)	C8—C9—C14—C13	−178.61 (17)

### Hydrogen-bond geometry (Å, °)

**Table:**

*D*—H···*A*	*D*—H	H···*A*	*D*···*A*	*D*—H···*A*
N3—H3A···N1^i^	0.90 (1)	1.96 (1)	2.815 (2)	158.(2)

Symmetry codes: (i) *x*+1/2, −*y*+1/2, *z*−1/2.
